# Risk Factors for Endoscopic Gastric Mucosal Lesions: Analysis of Lifestyle, Dietary, and Clinical Determinants in 361 Patients

**DOI:** 10.3390/life15091474

**Published:** 2025-09-19

**Authors:** Patrick-Lazăr-Dominik Chiciudean, Ana-Maria Filip, Sabrina-Nicoleta Munteanu, Cristian-Ioan Cîmpian, Simona Mocan, Monica Pantea, Anca Elena Negovan

**Affiliations:** 1 Oncology Department, Mureș County Clinical Hospital, 540140 Târgu Mureș, Romania; patrickcld@icloud.com; 2 Department of Clinical Science-Internal Medicine, “George Emil Palade” University of Medicine, Phamacy, Science, and Technology, 540139 Târgu Mureș, Romania; sabrinamunteanu12@gmail.com (S.-N.M.); cmonicapantea@gmail.com (M.P.); ancanegovan@yahoo.com (A.E.N.); 3 Obstetrics and Gynecology Department, Emergency County Hospital of Târgu Mureș, 540136 Târgu Mureș, Romania; cristicimpian@yahoo.com; 4 Pathology Department, Emergency County Hospital of Târgu Mureș, 540136 Târgu Mureș, Romania; slmocan@yahoo.com

**Keywords:** gastric lesions, *Helicobacter pylori*, smoking, NSAIDs, risk factors, ordinal regression

## Abstract

Background/Objectives: Gastric mucosal lesions represent a significant health burden, with *Helicobacter pylori* infection being the primary cause of chronic gastritis worldwide. However, the role of modifiable lifestyle factors in modulating the severity of gastric lesions remains incompletely characterized, particularly in Eastern European populations. This study aimed to analyze the relationship between dietary behaviors, smoking, alcohol consumption, and the severity of endoscopic gastric lesions in Romanian patients. Methods: We conducted a cross-sectional study including 361 patients who underwent upper gastrointestinal endoscopy at Târgu Mureș County Clinical Emergency Hospital between 2019 and 2025. Endoscopic lesion severity was classified on an ordinal scale (0 = normal; 1 = edema/erythema; 2 = erosions; 3 = ulcer/bleeding). Dietary intake was assessed using a validated food frequency questionnaire, with foods classified as pro-inflammatory or protective. Ordinal logistic regression models were used to examine associations between lifestyle factors and the severity of gastric lesions, adjusted for age, sex, and *H. pylori* status. Results: Among participants (median age 65 years, 46.5% male), 45.2% had clinically significant lesions (≥2). *H. pylori* infection was present in 31.6% of participants. Current smoking (15.2% of participants) showed a trend toward increased severity of gastric lesions (fully adjusted OR 1.59, 95% CI 0.93–2.71, *p* = 0.092), though not statistically significant. Among current smokers, 52.7% had clinically significant lesions versus 43.8% among non/former smokers. The smoking–alcohol interaction was not statistically significant (interaction OR = 1.19, 95% CI: 0.34–4.17, *p* = 0.780). Dietary balance score showed no association with the severity of gastric lesions (OR = 1.061 per 10-unit increase, *p* = 0.355). NSAID use emerged as the strongest predictor (OR = 1.68, 95% CI 1.01–2.78, *p* = 0.044). The number of cumulative risk factors correlated significantly with clinically significant lesions (Spearman r = 0.107, *p* = 0.042), with prevalence increasing from 34.5% in patients with 0–1 factors to 83.3% with 6+ factors. Conclusions: Current smoking showed a trend toward increased severity of gastric lesions in this Romanian cohort, though not reaching statistical significance. NSAID use was the only significant independent predictor. The dose–response relationship between cumulative risk factors and the severity of lesions emphasizes the importance of comprehensive risk assessment and multi-factorial interventions in gastric disease prevention. However, as a cross-sectional study, these associations cannot establish causality and should be confirmed in prospective cohorts.

## 1. Introduction

Gastric mucosal lesions identified during upper gastrointestinal endoscopy represent a spectrum of pathological changes ranging from mild erythema and superficial erosions to deep ulcers, submucosal hemorrhages, or changes suggesting glandular atrophy and intestinal metaplasia. While *H. pylori* infection remains the primary cause of chronic gastritis worldwide, recent evidence has highlighted the significant impact of modifiable lifestyle factors on gastric mucosal health [1,2,3].

The relationship between dietary patterns and gastric pathology has been extensively studied across different populations. High-salt diets and salt-preserved foods are strongly associated with gastric mucosal lesions, with epidemiological data showing higher rates of chronic gastritis, peptic ulcers, and gastric cancer in regions with elevated consumption of salted foods, particularly in East Asia and Eastern Europe [4,5]. Salt directly affects the protective mucous layer and facilitates *H. pylori* colonization, creating a synergistic effect that accelerates mucosal damage [6,7].

Processed meat consumption represents another significant dietary risk factor through its high content of nitrites and nitrates, which can form carcinogenic N-nitroso compounds in the acidic gastric environment [8,9,10]. Conversely, diets rich in fresh fruits and vegetables provide antioxidant vitamins and polyphenols that neutralize oxidative stress and may inhibit *H. pylori* growth [11,12,13].

Smoking is a well-established lifestyle factor that adversely affects the upper gastrointestinal tract. Recent large-scale studies have confirmed that smokers present higher rates of gastritis and peptic ulcer disease, with nicotine and tobacco chemicals reducing mucosal blood flow and bicarbonate secretion while promoting duodenogastric reflux [14,15,16]. Similarly, alcohol consumption, particularly when excessive or chronic, directly damages the mucosal barrier and triggers inflammatory pathways that compromise tissue repair [17,18].

Romania, as part of Eastern Europe, maintains *H. pylori* infection rates that, although decreasing, remain higher than in Western Europe [4]. Combined with alcohol consumption above the WHO European average, smoking prevalence of 34%, and traditional preservation methods still used in rural areas, these factors create a unique epidemiological context [19,20].

Despite the extensive international literature on gastric risk factors, there is limited data specifically addressing the Romanian population. The applicability of guidelines developed for Western European or Asian populations requires validation in local contexts, considering genetic, dietary, and socioeconomic differences. Furthermore, the interaction between multiple lifestyle factors and their cumulative effect on gastric mucosal severity remains incompletely characterized.

This study examined 361 Romanian patients to analyze the relationship between dietary behaviors, smoking, alcohol consumption, and the severity of endoscopic gastric lesions. We tested four hypotheses: whether smoking independently predicts clinically significant gastric lesions, whether alcohol potentiates smoking effects, how dietary patterns influence lesion severity, and whether risk factors show cumulative dose–response relationships. This integrated analysis provides evidence-based data essential for developing prevention strategies adapted to our population’s specific characteristics. While previous studies have established individual risk factors for gastric pathology, this study provides several contributions to the existing literature: (1) it analyses the interplay of multiple risk factors in a Romanian population through ordinal regression modeling, expanding the limited epidemiological evidence from Eastern European contexts; (2) it examines smoking–alcohol interactions in the context of regional consumption patterns characterized by high spirits intake; (3) it applies ordinal regression modeling to preserve the ordered nature of endoscopic severity, allowing for more nuanced analysis of dose–response relationships between risk factors and gastric mucosal lesions.

## 2. Materials and Method

### 2.1. Study Design and Participants

This cross-sectional study included 361 patients who underwent upper gastrointestinal endoscopy at Târgu Mureș County Clinical Emergency Hospital between 2019 and 2025. The study protocol was approved by the Medical Ethics Committee for Clinical Drug Research of Târgu Mureș County Clinical Emergency Hospital (Decision No. Ad. 15347), and all participants provided written informed consent.

Inclusion criteria were: (1) age ≥ 18 years; (2) clinical indication for upper endoscopy (dyspepsia, gastroesophageal reflux symptoms, anemia, or screening); (3) complete endoscopic visualization of gastric mucosa; (4) systematic gastric biopsies obtained per Sydney System protocol; (5) completed validated dietary assessment questionnaire; (6) available laboratory data including *H. pylori* status determined by histopathology. Patients were excluded if they had: (1) previous gastric surgery; (2) active gastrointestinal bleeding requiring immediate intervention; (3) known or suspected gastric malignancy; (4) severe systemic conditions potentially affecting gastric mucosa; (5) inadequate endoscopic visualization; or (6) incomplete data for primary outcome variables.

### 2.2. Clinical Data Collection

Demographic and clinical data were collected through structured interviews and medical record review. Variables included age, sex, current medications (specifically NSAIDs and proton pump inhibitors), smoking and alcohol consumption history. NSAID use was defined as regular intake (chronic use) of non-steroidal anti-inflammatory drugs including aspirin within the 4 weeks preceding endoscopy. Occasional use was categorized as non-use. Smoking status was categorized as follows: never smoker, former smoker (>10 years since cessation), former smoker (<10 years since cessation), current smoker (>10 years), and current smoker (<10 years) [21]. Alcohol consumption was assessed by recording the frequency and quantity of wine, beer, and spirits consumed per week. Standard units were calculated as follows: one unit per serving of wine, two units per serving of beer, and two units per serving of spirits, where one unit equals 10 g of pure alcohol [22].

### 2.3. Laboratory Assessments

Venous blood samples were collected for routine laboratory analyses. Hemoglobin levels and mean corpuscular volume (MCV) were measured using standard automated hematology analyzers. *Helicobacter pylori* status was determined by histopathological examination of gastric biopsies obtained during endoscopy. At least two biopsies were taken from the antrum and two from the corpus according to the Sydney System protocol.

### 2.4. Dietary Assessment

Dietary intake was evaluated using an adapted version of a validated semi-quantitative food frequency questionnaire assessing consumption of 22 food items over the previous month [23]. The questionnaire was administered by trained personnel during face-to-face interviews, with a standard portion size guide used to ensure consistency. Consumption frequency was recorded as portions per week.

Foods were classified as pro-inflammatory (white bread, pickled vegetables, sauces/condiments, French fries, pizza, pasta, salty snacks, sweets, non-smoked processed meats, smoked processed meats, ground meat, red meat, salty cheese, smoked fish) or protective (whole grain bread, fresh cheese, white meat, rice/cereals, fruits, vegetables) based on their documented effects on gastric mucosa.

Pro-inflammatory foods included the following: (1) high-salt items (pickled vegetables, salty snacks, salty cheese) shown to damage the mucosal barrier and facilitate *H. pylori* colonization [6,7]; (2) processed and smoked meats (including ground meat and smoked fish) containing nitrites/nitrates that form N-nitroso compounds [8,9,10]; (3) red meat associated with heme iron-induced oxidative stress and increased cancer risk [8,10]; (4) refined carbohydrates and high-glycemic foods (white bread, pasta, sweets, pizza) linked to increased inflammatory markers; (5) fried foods (French fries) containing trans fats and advanced glycation end products [24]; and (6) condiments/sauces often high in salt, sugar, and preservatives [25].

Protective foods included the following: (1) fruits and vegetables providing antioxidants (vitamins C, E, polyphenols) that neutralize oxidative stress and may inhibit *H. pylori* growth [11,12]; (2) whole grain bread providing fiber, B vitamins, and anti-inflammatory compounds [26]; (3) lean proteins (white meat, fresh cheese) with lower saturated fat content [13]; and (4) rice/cereals as complex carbohydrates with lower inflammatory potential compared to refined grains [27,28]. This classification aligns with Mediterranean dietary patterns shown to reduce gastric pathology risk [13]. Pro-inflammatory and protective diet scores were calculated as the sum of weekly portions for each category.

### 2.5. Endoscopic Assessment

Upper gastrointestinal endoscopies were performed by experienced gastroenterologists after overnight fasting using high-definition endoscopes (Olympus EVIS EXERA XI systems, Olympus Corp. Tokyo, Japan). The entire gastric mucosa was systematically examined with photo-documentation of pathological findings. We used a simplified modification of the Lanza score [29], condensing the original 5 grades into 4 categories for statistical efficiency: 0 = normal mucosa; 1 = edema/erythema; 2 = erosions (corresponding to Lanza grades 2–3); 3 = ulcer/bleeding (corresponding to Lanza grade 4), adapted to create broader categories suitable for our sample size and ordinal regression analysis. Preneoplastic lesions (atrophy, intestinal metaplasia) and angiodysplasias were documented separately but excluded from the severity of gastric lesions scoring as they represent different pathophysiological processes. Clinically significant lesions were defined as having a score ≥2 on the gastric lesion severity scale.

### 2.6. Statistical Analysis

Statistical analyses were performed using R version 4.3.0 (R Foundation for Statistical Computing, Vienna, Austria) with the following packages: tidyverse (v2.0.0) for data manipulation, gtsummary (v1.7.2) for descriptive statistics, MASS (v7.3-60) for ordinal logistic regression, and ggplot2 (v3.4.4) for visualization.

Continuous variables were assessed for normality using the Shapiro–Wilk test. Normally distributed variables were presented as mean (standard deviation) and compared using one-way ANOVA. Non-normally distributed variables were presented as median (interquartile range) and compared using the Kruskal–Wallis test. Categorical variables were presented as *n* (%) and compared using Fisher’s exact test. For analysis purposes, current and former smokers < 10 years were combined for some analyses due to small numbers in certain categories.

The primary analysis examined the association between smoking and the severity of gastric lesions using ordinal logistic regression with the proportional odds model (polr function, MASS package). Three models were constructed: Model 1 (unadjusted), Model 2 (adjusted for age and sex), and Model 3 (fully adjusted for age, sex, and *H. pylori* status). The proportional odds assumption was verified using the Brant test.

Secondary analyses included the following: (1) evaluation of smoking–alcohol interaction using multiplicative interaction terms in the regression model; (2) assessment of dietary factors using separate models for pro-inflammatory and protective diet scores; and (3) cumulative risk analysis examining the dose–response relationship between the number of risk factors and lesion severity using Spearman correlation.

All statistical tests were two-sided, and *p*-values < 0.05 were considered statistically significant. Only patients with complete data for all study variables were included in the final analysis (*n* = 361). Patients with any missing values were excluded during the enrollment phase. All *p*-values were reported to three decimal places. Baseline characteristics across endoscopic severity groups are presented in Table 1.

## 3. Results

### 3.1. Participant Characteristics

Among 361 participants, the median age was 65.0 years (IQR 55.0–73.0), and 168 (46.5%) were male. The distribution of endoscopic severity was follows: normal (*n* = 35, 9.7%), edema/erythema (*n* = 163, 45.2%), erosions (*n* = 87, 24.1%), and ulcer/bleeding (*n* = 76, 21.1%). Current smoking was reported by 55 participants (15.2%), while *H. pylori* infection was present in 114 (31.6%) participants (Table 1).

**Table 1:** Baseline characteristics of 361 patients stratified by endoscopic lesions severity.

		Endoscopic Severity				
Characteristic	All Patients (*n* = 361) ^1^	Normal *n* = 35 ^1^	Edema/Erythema *n* = 163 ^1^	Erosions *n* = 87 ^1^	Ulcer/Bleeding *n* = 76 ^1^	*p* Value ^2^
Age, years	65.0 (55.0, 73.0)	64.0 (53.0, 77.0)	64.0 (53.0, 70.0)	66.0 (57.0, 72.0)	70.0 (58.0, 77.5)	0.015
Sex						0.242
Female	193 (53)	22 (63)	91 (56)	39 (45)	41 (54)	
Male	168 (47)	13 (37)	72 (44)	48 (55)	35 (46)	
NSAIDs use	59 (16)	4 (11)	21 (13)	19 (22)	15 (20)	0.207
PPI	194 (54)	17 (49)	91 (56)	40 (46)	46 (61)	0.245
*H. pylori* infection						0.198
Negative	247 (68)	19 (54)	118 (72)	60 (69)	50 (66)	
Positive	114 (32)	16 (46)	45 (28)	27 (31)	26 (34)	
Smoking status						0.097
Never smoker	225 (62)	29 (83)	101 (62)	44 (51)	51 (67)	
Former smoker > 10 y	45 (12)	5 (14)	19 (12)	13 (15)	8 (11)	
Former smoker < 10 y	36 (10)	0 (0)	18 (11)	12 (14)	6 (8)	
Current smoker > 10 y	49 (14)	1 (3)	21 (13)	17 (20)	10 (13)	
Current smoker < 10 y	6 (2)	0 (0)	4 (2)	1 (1)	1 (1)	
Alcohol consumption, units/week	0.0 (0.0, 2.0)	0.0 (0.0, 1.0)	0.0 (0.0, 4.0)	0.0 (0.0, 4.0)	0.0 (0.0, 2.0)	0.389
Hemoglobin, g/dL	12.2 (2.7)	10.6 (2.8)	12.5 (2.5)	12.6 (2.8)	11.7 (2.9)	<0.001
Mean corpuscular volume, fL	87 (9)	89 (11)	86 (10)	87 (7)	87 (10)	0.555
Pro-inflammatory diet score	11.7 (8.4, 15.1)	13.0 (9.4, 14.3)	11.7 (8.2, 15.3)	12.2 (7.4, 16.3)	11.0 (8.3, 13.9)	0.559
Protective diet score	26.2 (20.8, 31.0)	26.2 (20.8, 31.5)	25.6 (20.8, 31.0)	26.8 (20.8, 31.0)	25.6 (20.8, 31.5)	0.996

^1^ Data are presented as median (interquartile range) for continuous variables and n (%) for categorical variables unless otherwise specified. Hemoglobin and mean corpuscular volume are presented as mean (standard deviation). ^2^ Statistical significance at *p* < 0.05. NSAIDs, non-steroidal anti-inflammatory drugs; PPI, proton pump inhibitor; *H. pylori*, *Helicobacter pylori*.

Patients with more severe lesions were older (*p* = 0.015, Table 1). Paradoxically, hemoglobin levels showed a non-linear pattern across severity groups (*p* < 0.001), with the lowest values observed in patients with endoscopically normal mucosa (10.6 g/dL) compared to those with edema/erythema (12.5 g/dL), erosions (12.6 g/dL), or ulcer/bleeding (11.7 g/dL). This unexpected finding suggests that patients with normal endoscopy may have been referred for investigation of unexplained anemia or other systemic conditions. The prevalence of *H. pylori* infection did not differ significantly across severity groups (*p* = 0.198).

### 3.2. Smoking as an Independent Risk Factor

**Figure 1:**
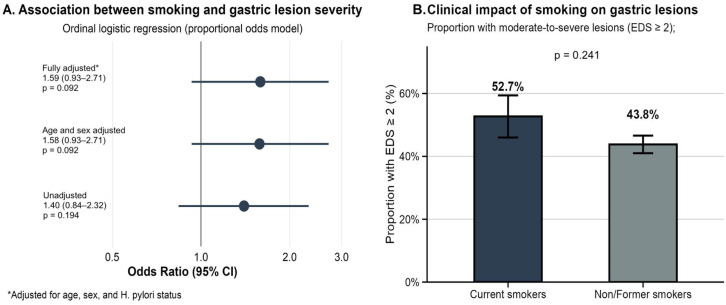
Association between smoking and the severity of gastric lesions. (**A**) Forest plot showing odds ratios and 95% confidence intervals from ordinal logistic regression models. (**B**) Proportion of patients with clinically significant lesions (≥2) by smoking.

In ordinal logistic regression analysis, current smoking was associated with increased odds of more severe endoscopic lesions, though this did not reach statistical significance (Figure 1A). The unadjusted OR was 1.40 (95% CI 0.84–2.32, *p* = 0.194). After adjustment for age and sex, the association strengthened slightly (OR 1.58, 95% CI 0.93–2.71, *p* = 0.092) and remained similar after additional adjustment for *H. pylori* status (OR 1.59, 95% CI 0.93–2.72, *p* = 0.092). The proportional odds assumption was satisfied (Brant test *p* = 0.456).

Current smokers had a higher proportion of clinically significant lesions (≥2) compared to non/former smokers (52.7% vs. 43.8%), though this difference was not statistically significant (*p* = 0.241, absolute risk increase 8.9%, NNH = 11) (Figure 1B).

### 3.3. Interaction Between Smoking and Alcohol Consumption

The formal test for interaction between current smoking and alcohol consumption (>7 units/week) was not statistically significant (interaction OR = 1.19, 95% CI: 0.34–4.17, *p* = 0.780). Among moderate/heavy drinkers (>7 units/week, *n* = 48), current smokers had more than twice the odds of severe lesions compared to non-smokers (OR = 2.21, 95% CI: 0.61–8.08, *p* = 0.230), although the confidence interval was wide due to limited sample size.

**Figure 2:**
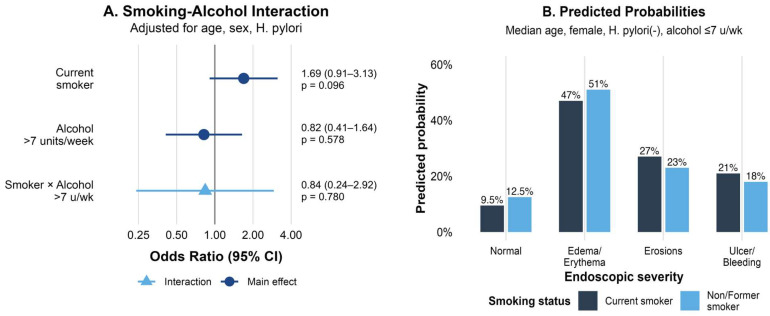
Smoking–alcohol interaction in the severity of gastric lesions. (**A**) Forest plot showing stratified odds ratios for current smoking by alcohol consumption level. (**B**) Proportion of patients with moderate-to-severe lesions (severity grade ≥ 2) across smoking and alcohol consumption categories.

Stratified analysis revealed differential effects of smoking by alcohol consumption status (Figure 2A). Among non/light drinkers (≤7 units/week), current smoking was associated with OR = 1.62 (95% CI: 0.88–2.99), while the effect appeared more pronounced among moderate/heavy drinkers. The distribution of endoscopic severity across combined smoking and alcohol categories showed the highest proportion of severe lesions in current smokers who consumed > 7 units/week (Figure 2B).

### 3.4. Dietary Balance and the Severity of Gastric Lesions

The study population consumed a mean of 23.8 ± 11.1 portions/week of pro-inflammatory foods and 22.2 ± 7.7 portions/week of protective foods, resulting in a slightly negative mean dietary balance score of −1.6 ± 15.2. Contrary to our hypothesis, the dietary balance score was not significantly associated with the severity of gastric lesions. Each 10-unit increase toward a more protective dietary balance was associated with a non-significant 6.1% increase in the odds of more severe lesions (OR = 1.061, 95% CI: 0.936–1.201, *p* = 0.355).

**Figure 3:**
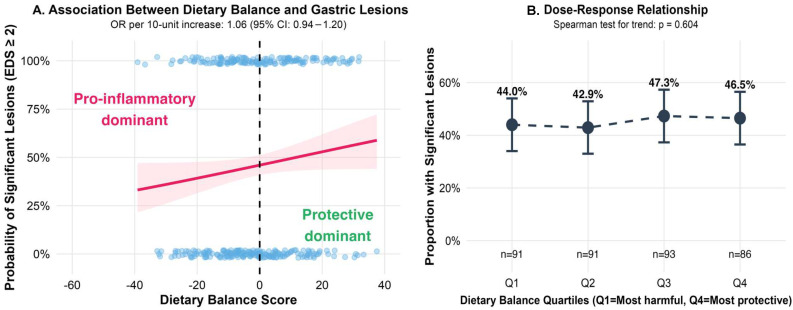
Association between dietary factors and the severity of gastric lesions. (**A**) Dose–response relationship between dietary balance score quartiles and proportion of patients with clinically significant lesions (severity grade ≥ 2); The red line shows the fitted logistic regression model with 95% confidence interval (pink shaded area). Blue dots represent individual participants. (**B**) Forest plot showing odds ratios for individual dietary components and the severity of gastric lesions.

Analysis by quartiles of dietary balance revealed no dose–response relationship (Spearman test for trend: *p* = 0.604), with similar distributions across severity groups (Figure 3A). The proportion of patients with clinically significant lesions (severity grade ≥ 2) was similar across quartiles, ranging from 42.9% in Q2 to 47.3% in Q3. When examining individual dietary components, forest plot analysis showed no significant associations between specific food groups and the severity of gastric lesions, with confidence intervals crossing unity for all items examined (Figure 3B).

### 3.5. Cumulative Risk Factors and Dose–Response Relationship

The distribution of risk factors showed considerable heterogeneity, with a mean of 2.73 ± 1.31 factors per patient. The most prevalent risk factors were advanced age (62.6%), elevated MCV (49.3%), male sex (46.5%), and anemia (44.0%). The ordinal logistic regression model revealed a non-significant trend toward increased lesion severity with each additional risk factor (OR = 1.057, 95% CI: 0.879–1.272, *p* = 0.555).

**Figure 4:**
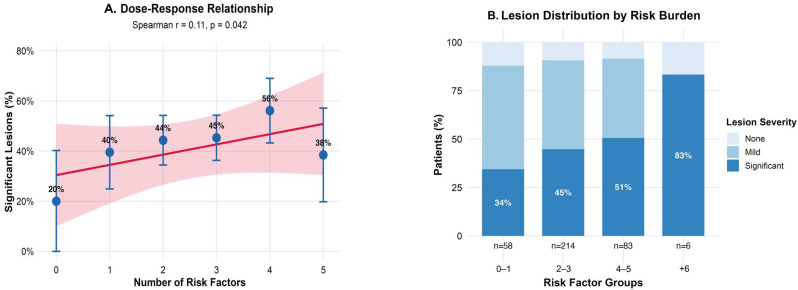
Cumulative risk factors and the severity of gastric lesions. (**A**) Dose–response relationship between number of risk factors and proportion of patients with clinically significant lesions (severity grade ≥ 2). (**B**) Forest plot showing odds ratios and 95% confidence intervals for individual risk factors from multivariate ordinal logistic regression analysis.

However, Spearman correlation analysis demonstrated a significant positive correlation between the number of risk factors and the presence of clinically significant lesions (r = 0.107, *p* = 0.042). The proportion of patients with significant lesions (≥2) showed a clear dose–response pattern (Figure 4A): 34.5% in those with 0–1 factors, 44.9% with 2–3 factors, 50.6% with 4–5 factors, and 83.3% with 6+ factors, representing a 2.4-fold increase from the lowest to highest risk group. Individual risk factor analysis (Figure 4B) identified NSAID use as the strongest independent predictor (OR = 1.68, 95% CI: 1.01–2.78, *p* = 0.044), followed by active smoking (OR = 1.58, 95% CI: 0.92–2.71, *p* = 0.092). No significant interaction was found between *H. pylori* infection and smoking status (*p* = 0.418).

## 4. Discussion

This study provides a comprehensive analysis of multiple lifestyle factors and their cumulative effect on the severity of endoscopic gastric lesions in a well-characterized Romanian cohort from Târgu Mureș.

Current smoking showed a clinically relevant trend toward increased severity of gastric lesions (OR 1.59, 95% CI 0.93–2.71, *p* = 0.092), with 50.9% of smokers having significant lesions versus 43.3% of non-smokers. While not statistically significant, this 7.6% absolute risk increase (NNH = 11) aligns with international meta-analyses reporting ORs of 1.5–2.0 for smoking-related gastric lesions [30,31,32,33]. The attenuated effect in our population may reflect the relatively low proportion of current smokers (15.2%) or potential underreporting. The dose–response relationship observed for long-term smokers supports cumulative tobacco toxicity on gastric mucosa.

Contrary to our hypothesis, we found no significant smoking–alcohol interaction (*p* = 0.780). Although current smokers who consumed > 7 units/week showed higher odds of severe lesions (OR 2.21), the wide confidence interval reflects limited power. This contrasts with other studies showing strong synergistic effects [34,35,36,37]. This unexpected finding is particularly intriguing given Romania’s high alcohol consumption patterns. According to WHO Global Status Report on Alcohol and Health 2018, Romania reports 12.6 L of pure alcohol per capita consumption annually, with heavy episodic drinking affecting 53.8% of male drinkers. The pattern is dominated by spirits, including unrecorded consumption of traditional țuică [38]. Despite this high-risk drinking pattern, which theoretically should potentiate smoking effects, we observed no significant interaction.

Several factors may explain this paradox. First, our alcohol measurement (units/week) may not adequately capture Romania’s episodic heavy drinking pattern. Second, Romania’s mixed alcohol consumption pattern (46% beer, 31% spirits including unrecorded țuică) differs from the more homogeneous drinking patterns in populations where synergistic effects have been documented [35,39]. Third, genetic polymorphisms affecting alcohol metabolism may differ between Romanian and Western populations, potentially modulating smoking–alcohol interactions differently.

NSAID use emerged as the strongest independent predictor of gastric lesions (OR 1.68, 95% CI 1.01–2.78, *p* = 0.044), consistent with international data [40,41,42]. With 16.3% of our cohort using NSAIDs, this finding has immediate clinical implications for gastroprotective co-prescription strategies.

An unexpected finding was the apparent paradoxical relationship between hemoglobin levels and endoscopic severity, with the lowest mean values observed in patients with endoscopically normal mucosa (10.6 g/dL) compared with those presenting erosions (12.6 g/dL, *p* < 0.001). This likely reflects selection bias, as patients with normal-appearing mucosa may have been referred primarily for the investigation of unexplained anemia, whereas those with visible lesions were more often investigated for dyspeptic symptoms rather than anemia. The intermediate hemoglobin level in the ulcer group (11.7 g/dL) might be attributable to prior iron supplementation in patients with known peptic disease. These findings emphasize that a macroscopically normal endoscopy does not exclude gastrointestinal causes of anemia and highlight the limitations of endoscopic severity scores in capturing the full spectrum of gastric pathology with systemic impact.

The significant dose–response relationship between cumulative risk factors and the severity of gastric lesions (Spearman r = 0.107, *p* = 0.042) supports a multifactorial disease model. The prevalence of significant lesions increased from 34.5% with 0–1 risk factors to 83.3% with 6+ factors, emphasizing the value of comprehensive risk assessment.

Surprisingly, *H. pylori* infection showed no association with the severity of gastric lesions despite 31.6% prevalence (*p* = 0.198). The highest *H. pylori* prevalence paradoxically occurred in patients with normal mucosa (45.7%) versus those with ulcer/bleeding (34.2%). This pattern may reflect successful eradication in previously treated symptomatic patients, strain-specific factors not captured in our analysis, or the “African enigma” phenomenon where high *H. pylori* prevalence does not correlate with expected pathology [43].

Our study’s strengths include a systematic and standardized endoscopic assessment covering the full spectrum of gastric lesion severity, the use of ordinal regression modeling that preserved the ordered nature of severity scores, and the inclusion of an understudied Eastern European population with distinctive risk factor profiles. These aspects enhance both the methodological rigor and the relevance of our findings for similar settings. Limitations include the cross-sectional design, which precludes causal inference, and potential selection bias as only symptomatic patients undergoing upper endoscopy were included. The concurrent use of proton pump inhibitors (54% of participants) represents a potential confounding factor that may have masked more severe mucosal lesions and influenced the observed associations. Histopathological correlation was not systematically performed for all patients with lesions classified as severity grade 3, which represents a limitation in validating the endoscopic severity scoring against microscopic pathology. The statistical power was limited for certain subgroup analyses, leading to wide confidence intervals in some estimates. Additionally, data on *H. pylori* virulence factors (CagA/VacA), coffee consumption, stress levels, and genetic polymorphisms were not available, which may have limited the ability to fully adjust for these potential confounders. Another limitation is the absence of detailed quantitative measures of smoking exposure (e.g., pack-years) and refined characterization of alcohol consumption patterns (e.g., binge drinking versus regular moderate intake). This limited our ability to investigate dose–response relationships and may have contributed to the wide confidence intervals observed in subgroup analyses. Future studies should include: (1) propensity score matching to balance baseline characteristics between exposure groups; (2) longitudinal follow-up of patients with severity grade ≥ 2 to assess progression and response to risk factor modification; (3) histopathological correlation for all grades of gastric lesion severity, particularly for detecting preneoplastic changes; (4) stratified analysis by PPI use to clarify its modulating effects; and (5) more granular lifestyle exposure metrics to better capture dose–response associations.

These findings support integrated prevention strategies targeting multiple modifiable factors simultaneously. Clinical priorities should include routine NSAID risk assessment with gastroprotection when indicated, smoking cessation counseling that emphasizes the gastrointestinal as well as systemic health benefits, and enhanced surveillance for patients presenting with four or more concurrent risk factors. Future research should prospectively evaluate whether risk factor modification reduces lesion progression and should design region-specific prediction models that account for the high alcohol consumption patterns characteristic of this population.

## 5. Conclusions

NSAID use emerged as the strongest independent predictor of gastric mucosal lesions in this endoscopic population, highlighting the importance of gastroprotective strategies in at-risk populations. While current smoking showed a clinically relevant trend toward increased lesions severity, this association was not significant in our low-prevalence smoker cohort, and no significant association was observed with dietary pattern.

The significant dose–response relationship between cumulative risk factors and lesions severity emphasizes the multifactorial nature of gastric pathology. The lowest values of hemoglobin in endoscopically normal patients suggests important selection bias in endoscopic populations and highlights limitations of macroscopic severity scores.

These findings support integrated prevention strategies prioritizing NSAID risk assessment with appropriate gastroprotection, smoking cessation counseling, and enhanced surveillance for patients with multiple concurrent risk factors. Future prospective studies should evaluate whether targeted modification of these risk factors, particularly NSAID use and smoking, reduces progression of gastric mucosal lesions in high-risk Eastern European populations.

## Data Availability

The data presented in this study are available on reasonable request from the corresponding author. The data are not publicly available due to privacy and ethical restrictions related to patient confidentiality.

## Abbreviations

*H. pylori*	*Helicobacter pylori*
NSAID	Non-Steroidal Anti-Inflammatory Drug
MCV	Mean Corpuscular Volume
OR	Odds Ratio
CI	Confidence Interval
NNH	Number Needed to Harm
IQR	Interquartile Range
